# Increased lung inflammation with oxygen supplementation in tracheotomized spontaneously breathing rabbits: an experimental prospective randomized study

**DOI:** 10.1186/1471-2253-14-86

**Published:** 2014-10-01

**Authors:** Humberto S Machado, Catarina S Nunes, Paula Sá, Antonio Couceiro, Álvaro Moreira da Silva, Artur Águas

**Affiliations:** Serviço de Anestesiologia, Centro Hospitalar do Porto, Largo Abel Salazar, Porto, 4099-001 Portugal; Departamento de Ciências e Tecnologia, Universidade Aberta, Rua da Escola Politécnica 141, Lisboa, 1269-001 Portugal; Serviço de Anatomia Patológica, Centro Hospitalar Gaia/Espinho, Rua Conceição Fernandes, Vila Nova de Gaia, 4430 Portugal; Serviço de Cuidados Intensivos, Centro Hospitalar do Porto, Largo Abel Salazar, Porto, 4099-001 Portugal; Departamento de Anatomia Normal, Instituto Ciências Biomédicas Abel Salazar - Universidade do Porto, Rua Jorge Viterbo Ferreira, 228, Porto, 4050-313 Portugal; Unidade Multidisciplinar de Investigação Biomédica, Rua Jorge Viterbo Ferreira, 228, Porto, 4050-313 Portugal

**Keywords:** Lung inflammation, Oxygen supplemented spontaneous breathing

## Abstract

**Background:**

Mechanical ventilation is a well–known trigger for lung inflammation. Research focuses on tidal volume reduction to prevent ventilator-induced lung injury. Mechanical ventilation is usually applied with higher than physiological oxygen fractions. The purpose of this study was to investigate the after effect of oxygen supplementation during a spontaneous ventilation set up, in order to avoid the inflammatory response linked to mechanical ventilation.

**Methods:**

A prospective randomised study using New Zealand rabbits in a university research laboratory was carried out. Rabbits (n = 20) were randomly assigned to 4 groups (n = 5 each group). Groups 1 and 2 were submitted to 0.5 L/min oxygen supplementation, for 20 or 75 minutes, respectively; groups 3 and 4 were left at room air for 20 or 75 minutes. Ketamine/xylazine was administered for induction and maintenance of anaesthesia. Lungs were obtained for histological examination in light microscopy.

**Results:**

All animals survived the complete experiment. Procedure duration did not influence the degree of inflammatory response. The hyperoxic environment was confirmed by blood gas analyses in animals that were subjected to oxygen supplementation, and was accompanied with lower mean respiratory rates. The non-oxygen supplemented group had lower mean oxygen arterial partial pressures and higher mean respiratory rates during the procedure. All animals showed some inflammatory lung response. However, rabbits submitted to oxygen supplementation showed significant more lung inflammation (Odds ratio = 16), characterized by more infiltrates and with higher cell counts; the acute inflammatory response cells was mainly constituted by eosinophils and neutrophils, with a relative proportion of 80 to 20% respectively. This cellular observation in lung tissue did not correlate with a similar increase in peripheral blood analysis.

**Conclusions:**

Oxygen supplementation in spontaneous breathing is associated with an increased inflammatory response when compared to breathing normal room air. This inflammatory response was mainly constituted with polymorphonuclear cells (eosinophils and neutrophils). As confirmed in all animals by peripheral blood analyses, the eosinophilic inflammatory response was a local organ event.

## Background

Patients submitted to mechanical ventilation are at risk of developing acute respiratory distress syndrome (ARDS), either by overdistension (volutrauma) or repetitive tidal recruitment of closed lung units (atelectrauma) [1]. Overinflation of the more distal airways and alveoli is thought to be caused by high tidal volumes. The cycle of continuous opening and closing of alveolar groups also causes alveolar wall lesion and surfactant depletion, with injury by recruitment-derecruitment [2]. Continuous mechanical distension of type II alveolar cells contributes to an inflammatory response by changing the balance of pro and anti-inflammatory mediators [3]. Ventilation strategies near physiological values benefit patients from these conditions [1]. Post-operative lung inflammation and ARDS may also appear in less severe forms [4, 5], especially when overinflation is relevant [6]; however other pro-inflammatory factors may be present [7].

Oxygen has been characterized as a pro-inflammatory factor. Supra-physiological oxygen concentrations, as in mechanical ventilation, have been implied as a contributing factor to the development of lung injury; as described by Britt et al., newborn mice exposed to hyperoxia developed lung inflammation linked to cyclooxigenase pathways [8]. Lung injury by oxygen may occur as a result of injurious oxygen by-products (reactive oxygen species, ROS), and other key mediators (interferon-gama) secondary to hyperoxia [9, 10]. The type of oxygen toxicity to the lung has been found to be due to natural killer cell’s activation and proliferation in hyperoxic environments [11]; several levels of hyperoxia may play different roles on lung inflammation [12, 13].

Nevertheless, available evidence shows that hypoxia [4, 14] may also play an import role in the development of lung inflammation. If high oxygen content is applied to the lung, reabsorption atelectasis may develop, with resulting local hypoxemic environments; this fact may lead to a regional acidic milieu that results in subsequent inflammatory response [4]. Furthermore, continued hypoxia leads to an increased level of circulating pro-inflammatory cytokines and vascular leakage causing lung edema [14].

We hypothesized that supplementary oxygen may lead to enhanced lung inflammation, as a sole contributing factor. Therefore, we studied an animal model based on a spontaneous ventilation mode so as to avoid any bias from mechanical ventilation. Furthermore, exposure time to supplementary oxygen was also studied.

## Methods

### Animals

The experimental protocol used in this study was approved by appropriate local ethic committee (“Comité de Ética do ICBAS/UP”), and was done according to the European Union Directive n° 63/2010/EU. Twenty adult New Zealand rabbits (*Oryctolagus cuniculus*) were purchased from a Portuguese breeder (NORLAP – Rui M.S. Gonçalo, 4825–466 Água-Longa, Portugal) and were kept under standard housing conditions with unrestricted access to food and water, attended by veterinary doctors and inspected daily for wellbeing monitoring.

### Study groups

This study was conducted as prospective randomized animal experiment; we studied four groups with five rabbits each. Groups 1 and 2 two had supplementary oxygen with 20 and 75 minutes procedure respectively; Groups 3 and 4 had no supplementary oxygen with the same procedure duration (20 and 75 min). No controlled ventilation was used in any group.

### Instrumentation

All rabbits (mean weight of 2057 grams [g], std 227.19 g) were submitted to anesthesia with ketamine (100 milligrams [mg]/milliliter [ml] - Imalgene®, Bayer - Puteaux, France) and xylazine (20 mg/ml - Rompum®, Bayer HealthCare, Bayer Inc., Animal Health Division, Toronto, Canadá), with an initial dose 0.5 ml/kilogram [kg] of each of these drugs, supplemented intramuscularly with half dose every 20 minutes; animals were placed in the supine position and were kept this way during the whole procedure. Subsequently, the anterior region of the neck and abdomen were shaved and a 2 centimeter [cm] vertical midline incision was made on the anterior cervical region; after exposing the trachea, an incision was made so as to allow the introduction of a 2.5 tracheal tube to be connected to a spontaneous breathing system (Jackson-Rees type), with a fresh flow of oxygen of 0.5 liters [l] per minute in the oxygen supplemented group; no oxygen supplement was used in the non-supplemented group.

### Monitoring

Non-invasive monitoring was used to monitor heart rate, (nellcor oximax N600X®, Covidien, Boulder, CO, USA), rectal temperature and respiratory rate; values were collected every ten minutes after finishing the experimental setup. After tracheal intubation and supplementary oxygen in groups 1 and 2, an infra-abdominal incision was performed in all rabbits to allow aortic blood sample collection; these were collected at the beginning and just before termination of the procedure; blood was further analyzed for arterial blood gas and peripheral blood cell count determination.

### Tissue sampling

At the end of procedure all rabbits were euthanized with ketamine - xylazine 1 ml/kg of each drug, followed by section of abdominal aorta for exsanguination.

Rabbit lungs were harvested and fixed in 10% formaldehyde and embedded in paraffin, for light microscopy (LM) study. Three micra sections of each lung lobe (superior, middle and lower) were obtained from all rabbits. Lung sections were stained in hematoxylin and eosin.

### Histopathology

An arbitrarily defined 4 level inflammatory score was used to classify the lungs according to the intensity of inflammatory infiltrates in each lung plate: no inflammation, light, moderate and severe; light inflammation indicates infrequent inflammatory cells and/or inflammation confined to a few areas [15] (corresponding to less than 15 cells per high magnification field 40×). Moderate inflammation was defined as multiple areas in the tissue or a large area of inflammatory cells [15] (corresponding to an average of 16 to 25 cells per high magnification field 40×). Large multifocal areas of tissue with inflammatory cells or almost all areas of tissue affected were defined as severe inflammation [15] (corresponding to more than 25 cells per high magnification field 40×). Thus, rabbits were classified as light, moderate or severe inflammatory responders according to the intensity of inflammation assessed. Staff investigators in the animal laboratory were aware of group assignments. After laboratory lung harvesting and plate preparation, these were sent to an independent pathologist for histological scoring. The pathologist was unaware of group assignments. The results founded were accepted as valid.

It was also planned that if the duration of procedure did not influence the degree of inflammation, group 1 (n = 5) and group 2 (n = 5) would be joined, as *oxygen supplemented group* (OSG) (n = 10), and group 3 (n = 5) and group 4 (n = 5) joined in a *non-oxygen supplemented group* (NOSG) (n = 10) in a similar way.

### Statistics

The Resource Equation method [16] was used to determine if the sample size was appropriate for the experiment. A sample size of 5 rabbits per group with 4 groups (total n = 20) was considered adequate for the questions being asked, resulting in an adequate error component (df) between 10 and 20. Odds ratio (OR) was used as a measure of association between the inflammatory outcome and the study groups (95% confidence interval - CI). Kruskal-Wallis non-parametric test was used to compare variables between groups. Friedman test (Nonparametric two-way ANOVA) was used to compare variations within groups. A p-value < 0.05 was considered statically significant. The IBM SPSS Statistics 21 and Statistics Matlab® Toolbox from Mathworks were used for all calculations. Data is presented with median and range variables.

## Results

### Lung inflammation

All groups showed signs of moderate lung inflammation. Rabbits in Groups 1 and 2 were equally likely to have moderate inflammation (OR = 1). The same was found in group 3 and 4 (OR = 1) rabbits. Therefore, it was possible to aggregate the rabbits into two groups, those who had supplementary oxygen, (n = 10), and those who did not have supplementary oxygen (n = 10), since procedure duration did not influence the occurrence of inflammatory response. Thus, subsequent analysis was done considering only two groups, with or without oxygen supplement, OSG and NOSG, respectively.

**Figure 1:**
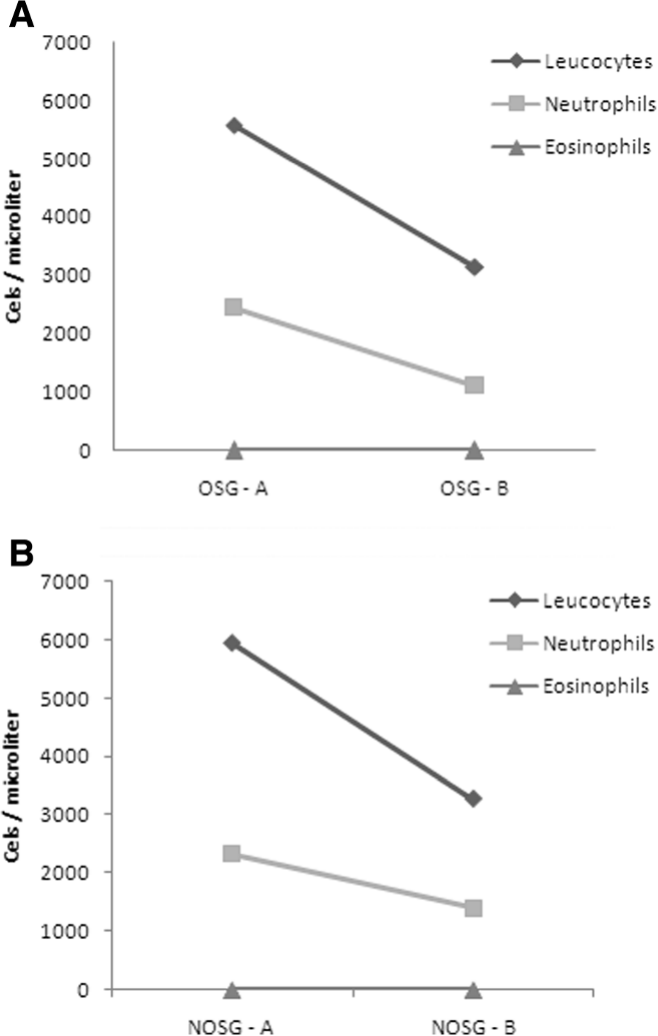
**Peripheral white cell count.** Legend: **(A)** first measurement, **(B)** final measurement at end of procedure, in OSG – oxygen supplemented group and in NOSG - non-oxygen supplemented group.

**Figure 2:**
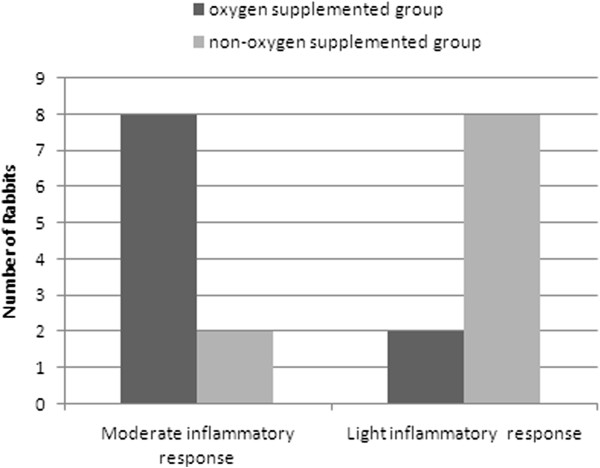
**Lung inflammatory response.** Moderate and mild inflammatory response in the OSG - oxygen supplemented group (n = 10) and NOSG - non-oxygen supplemented group (n = 10) (OR = 16).

**Figure 3:**
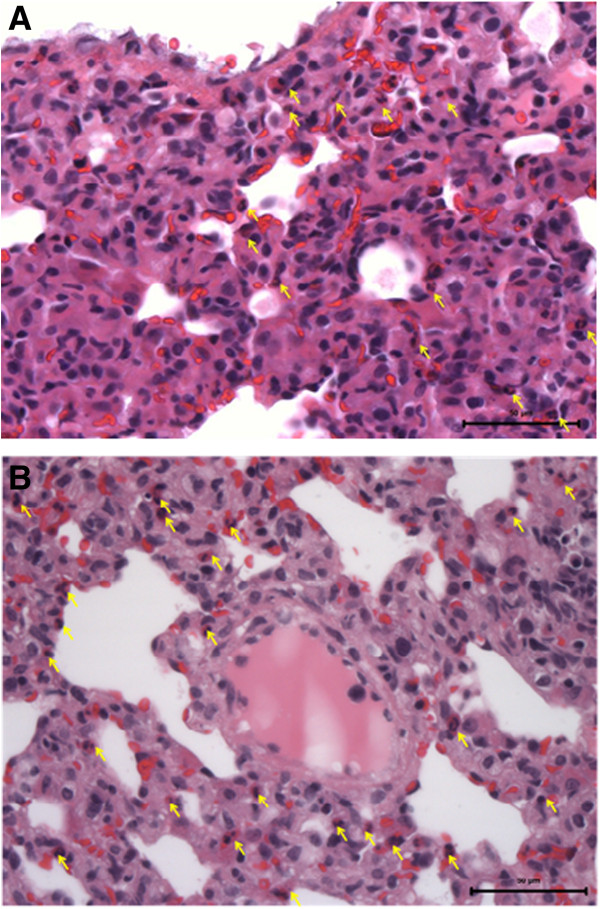
**Inflammatory cells.** **A** – Light inflammation, **B** – Moderate inflammation, Arrows - eosinophils.

The first median values of peripheral cell count measurement are shown in Figure 1. OSG showed moderate inflammation in 8 out of 10 subjects, whereas NOSG only did so in 2 rabbits, with an OR = 16 (CI 1.8-143.2); therefore OSG is more likely to have moderate inflation than the other group (Figure 2). The inflammatory cells found in these histological plates were neutrophils and eosinophils; with a proportion of about 80% eosinophils and 20% neutrophils (Figure 3).

### Heart rate, ventilatory rate, temperature and oxygen levels during the experiment

**Figure 4:**
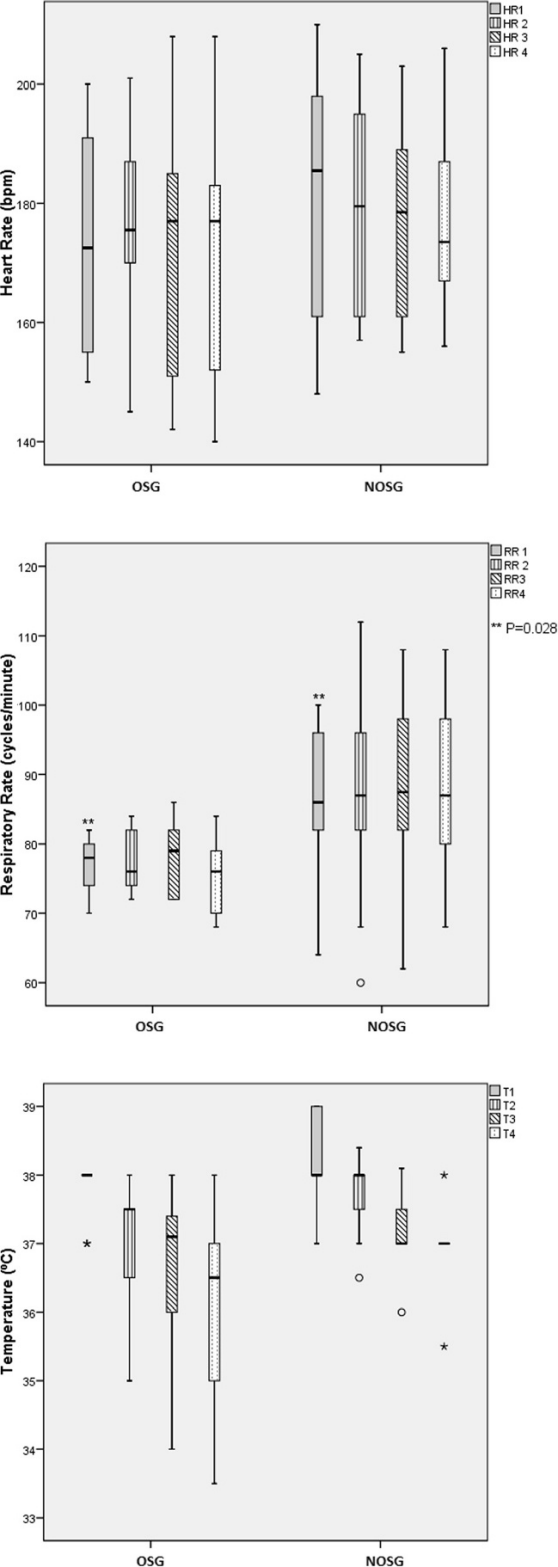
**Physiological parameters during procedure.** Median measures of heart rate, respiratory rate and temperature in OSG - oxygen supplemented group and NOSG - non-oxygen supplemented group, during procedure.

All animals survived the experiments and euthanasia was performed in order to collect lung samples. The median heart rate on first measurement was not different between groups (Figure 4) and did not change significantly during the procedure. The median respiratory rate (first measurement) was different between groups (Figure 1) (P = 0.028), but in both groups did not change significantly during the procedure. First measurement values for temperature were not statistically different between the two groups, but decreased significantly during procedure (P < 0.001) (Figure 4). The median rectal temperature (first measurement) was 38 degrees Celsius in the OSG and 38 degrees Celsius in the NOSG. In the OSG the median temperature decreased -3.9%, and in the NOSG the temperature decreased -2.6% (this decrease was not statistically different between groups).

**Figure 5:**
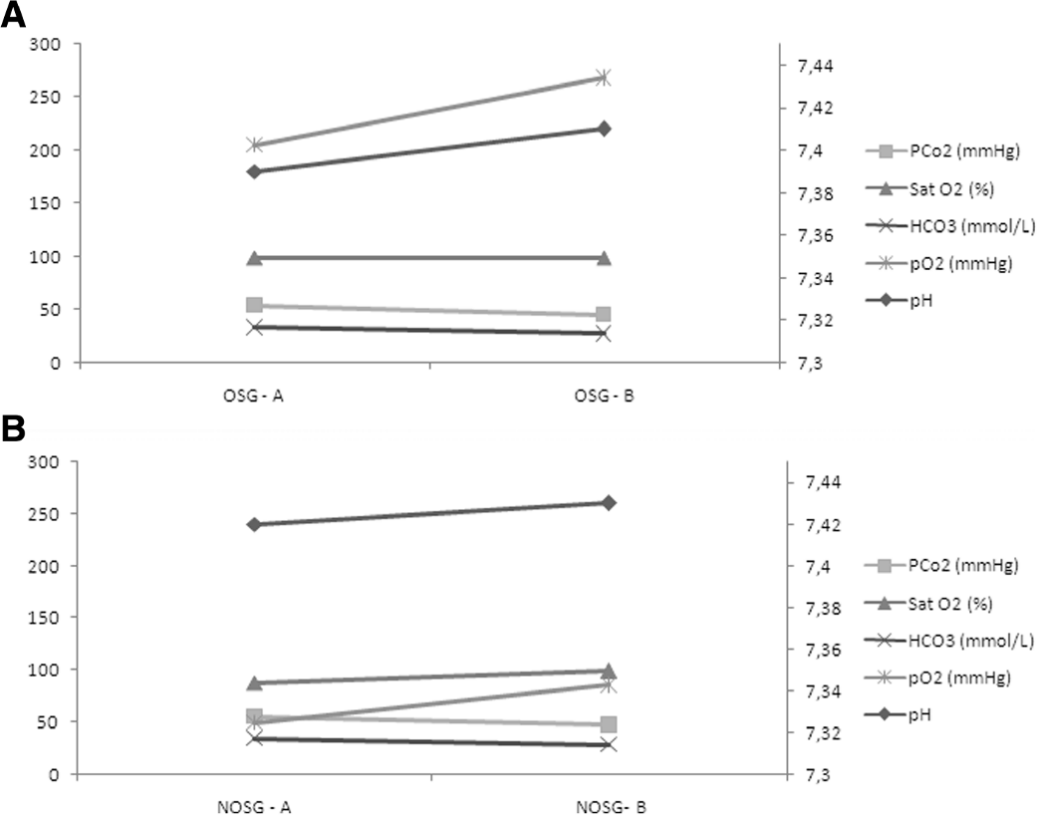
**Arterial blood gas analysis.** Arterial blood gas analysis at beginning of procedure **(A)** and at end of procedure **(B)** in OSG - oxygen supplemented group and NOSG - non-oxygen supplemented group.

A significant difference was found between the two groups in the first measurement values of arterial blood gas, namely partial oxygen pressure (pO2) and peripheral arterial saturation (Sat O2). At the end of the procedure the median pO2 increased by 8.89% in the OSG, and by 81.33% in the NOSG, this change was significantly different (P = 0.041). Sat O2 did not change (between first and final measurement) for the OSG, but it increased significantly by 13.36% in the NOSG (Figure 5).

## Discussion

Our data showed that the observed inflammatory response was more pronounced in the oxygen supplemented group, with a hyperoxic environment, a fact also found in other studies [17–19]; however it was predominantly constituted of eosinophils (80%) and to a minor extent by neutrophils (20%). This observation of the cellular content of the acute inflammatory response was unexpected; nevertheless, some evidence presented in literature might give some reasonable explanation for this occurrence. The common cells found in studies in which acute inflammatory response is observed are usually neutrophils and/or monocytes/macrophages [20–22]; however, the myeloid series implication on inflammation has been linked to a hypoxic model, with lactate accumulation and metabolic acidosis [22]. It is recognized that the eosinophil is armed with weapons able to kill helminthes and to damage tissues and cause disease [23]. In addition, eosinophils exhibit some properties that may support why we found them as the first representatives of acute inflammatory response [23]. These characteristics include the ability to migrate through endothelial cells without degranulation and without inducing vasculitis, its remarkable adherence capacity after minimal rolling to change it’s shape, and very quick spreading on the endothelial cell monolayer [23].

Moreover, one could question the animals’ initial health status, and an eventual eosinophil infiltration with a systemic expression. In regard to this, peripheral blood cell analysis was consistent with a healthy rabbit population in both groups, with special importance to the near absent peripheral eosinophil count (Figure 1). This fact draws away from the possibility of an eosinophil diseased rabbit population, which could be responsible for the lung infiltrates found.

Despite the more pronounced acute inflammatory infiltrate found in the OSG, with eosinophil predominance, this might seem to conflict with data showing hypoxia as a modulator of human eosinophil function [24]. This modulation might be expressed as an up-regulation on their survival [24]. However, the local consequence of oxygen supplementation is a hypoxic acidic environment, due to alveolar collapse arising from hyperoxia reabsorption atelectasis.

In our study, oxygen supplemented spontaneous ventilated animals had more pronounced inflammatory response than non-supplemented subjects, without influence from procedure duration. This fact is reasonably well documented [17–19], since oxygen is known as a possible trigger of inflammation. The association of controlled ventilation and hyperoxia are known as key synergetic factors responsible for ventilator induced lung injury (VILI) [20]. Our data in this spontaneous breathing model represents new insight in this matter, since previous known evidence (also in rabbits) shows that short term models for controlled ventilation might not trigger significant inflammatory responses [25]. As stated, we wanted to study the oxygen supplementation impact in a spontaneous model; the data presented showed that, even in this setting, oxygen itself plays one role in triggering inflammation as compared to room air ventilation.

Thus, it is reasonable to assume that as long as oxygen was used (even in a reduced exposure time of 20 minutes) in this spontaneous ventilation animal model, inflammation was immediately triggered and further enhanced as a moderate response. Potential atelectasis formation due to oxygen supplementation is widely described in literature [4]. This fact might explain a more pronounced inflammatory response in this group, because atelectasis correspond to a local phenomenon that induces local hypoxia and subsequent inflammatory signaling [4], with clear modification on cells life cycles and properties [8].

Being oxygen a known induction agent of absorption atelectasis, and subsequent generator of local acid inflammation in tissues, it might be reasonable to assume that larger areas of atelectasis might be linked to higher degrees of eosinophil infiltrates. However, further studies are needed to fully establish this correlation.

First measurement differences on respiratory rate that were found may be related to the fact that the OSG also had better pO2 values; thus, it is reasonably fair to assume that initial higher mean respiratory rate in NOSG may be due to extra effort in compensating transient initial lower pO2 (Figure 2). These facts were expected, and the experiment evolution proved that these higher respiratory rates from NOSG increased the pO2, a finding also confirmed (Figure 5).

## Conclusions

Oxygen sustainably enhances eosinophil based acute inflammatory response in the rabbit lung parenchyma in spontaneous ventilation mode, this when compared to a normoxic spontaneous ventilated group.

The time variable does not seem to influence the occurrence of the more pronounced inflammatory response (moderate); the main factor that appears to have influence is the presence or absence of oxygen supplementation.

## Authors’ information

Our multidisciplinary research group has several medical doctors with different specialties, namely anesthesiology (HM and PS), intensive care/chest medicine (AS) and pathology (AC), a mathematician (CN), and a senior research director of university laboratory (AA).

Our research has a specially designed animal model and set up (rabbit - *Oryctolagus cuniculus*) for investigating oxygen influence on ventilation in several ventilation modes. Ventilation has been a special interest of some of the authors (HM, PS, and AS) due to their routine clinical activity in operating room and intensive care. Assuming the challenges of controlled ventilation, the authors decided to investigate some variables that may enhance its risk, namely oxygen inspired fraction. To avoid mechanical ventilation bias, a spontaneous ventilation model was developed to overcome this problem. Proficiency in histological observation was guaranteed by a pathologist (AC) with a devoted professional career to lung pathology. Other studies are being planned to research related issues.

## References

[1] Hemmes SNT, Paulus F, Schultz MJ (2013). From the dark side of ventilation toward a brighter look at lungs. Crit Care Med.

[2] Hall NG, Liu Y, Hickman-Davis JM, Davis G, Myles C, Andrews EJ, Matalon S, Lang JD (2006). Bactericidal function of alveolar macrophages in mechanically ventilated rabbits. Am J Respir Cell Mol Biol.

[3] Hammerschmidt S, Kuhn H, Sack U, Schlenska A, Gessner C, Gillissen A, Wirtz H (2005). Mechanical stretch alters alveolar type II cell mediator release toward a proinflammatory pattern. Am J Respir Cell Mol Biol.

[4] Kilpatrick B, Slinger P (2010). Lung protective strategies in anaesthesia. Br J Anaest.

[5] Guarracino F, Baldassar R (2012). Perioperative acute lung injury: reviewing the role of anesthetic management. J Anesthe Clinical Res.

[6] Schilling T, Kozian A, Huth C, Buhling F, Kretzschmar M, Welte T, Hachenberg T (2005). The pulmonary immune effects of mechanical ventilation in patients undergoing thoracic surgery. Anesth Analg.

[7] De Conno E, Steurer MP, Wittlinger M, Zalunardo MP, Weder W, Schneiter D, Schimmer RC, Klaghofer R, Neff TA, Schmid ER, Spahn DR, Z’graggen BR, Urner M, Beck-Schimmer B (2009). Anesthetic-induced improvement of the inflammatory response to one-lung ventilation. Anesthesiology.

[8] Britt RD, Velten M, Tipple TE, Nelin LD, Rogers LK (2013). Cyclooxygenase-2 in newborn hyperoxic lung injury. Free Radic Biol Med.

[9] Iliodromiti Z, Zygouris D, Sifakis S, Pappa KI, Tsikouras P, Salakos N, Daniilidis A, Siristatidis C, Vrachnis N (2013). Acute lung injury in preterm fetuses and neonates: mechanisms and molecular pathways. J Matern Fetal Neonatal Med.

[10] Yamada M, Kubo H, Kobayashi S, Ishizawa K, Sasaki H (2004). Interferon-G: a key contributor to hyperoxia-induced lung injury in mice. Am J Physiol Lung Cell Mol Physiol.

[11] Nowak-Machen M, Schmelzle M, Hanidziar D, Junger W, Exley M, Otterbein L, Wu Y, Csizmadia E, Doherty G, Sitkovsky M, Robson SC (2013). Pulmonary NKT cells play an essential role in mediating hyperoxic acute lung injury. Am J Respir Cell Mol Biol.

[12] Waisman D, Brod V, Rahat MA, Amit-Cohen B, Lahat N, Rimar D, Menn-Josephy H, David M, Lavon O, Cavari Y, Bitterman H (2012). Dose related effects of hyperoxia on the lung inflammatory response in septic rats. Shock.

[13] Rogers LK, Tipple TE, Nelin LD, Welty SE (2009). Differential responses in the lungs of the new-born mouse pups exposed to 85% or >95% oxygen. Pediatr Res.

[14] Eltzschig HK, Carmeliet P (2011). Hypoxia and inflammation. N Engl J Med.

[15] Lamagna C, Scapini P, A. van Ziffle J, DeFranco AL, Lowell CA (2013). Hyperactivated MyD88 signaling in dendritic cells, through specific deletion of Lyn kinase, causes severe autoimmunity and inflammation. PNAS.

[16] Mead R (1988). The Design of Experiments.

[17] Lee PJ, Alam J, Sylvester W, Inamdar N, Otterbein L, Choi AMK (1996). Regulation of heme oxygenase-1 expression in vivo and in vitro in hyperoxic lung injury. Am J Respir Cell Mol Biol.

[18] Horinouchi H, Wang CC, Shepherd KE, Jones R (1996). TNF gene and protein expression in alveolar macrophages in acute and chronic hyperoxia-induced lung injury. Am J Respir Cell Mol Biol.

[19] Suzuki Y, Nishio N, Takeshita K, Takeuchi O, Watanabe K, Sato N, Naoki K, Kudo H, Aoki T, Yamaguchi K (2000). Effect of steroid on hyperoxia-induced ICAM-1 expression in pulmonary endothelial cells. Am J Physiol Lung Cell Mol Physiol.

[20] Quin DA, Moufarrej RK, Volokhov A, Hales CA (2002). Interactions of lung stretch, hyperoxia, and MIP-2 production in ventilator-induced lung injury. J Appl Physiol.

[21] Wilson MR, O’Dea KP, Zhang D, Shearman AD, Van Rooijen N, Takata M (2009). Role of lung-marginated monocytes in an in vivo mouse model of ventilator-induced lung injury. Am J Respir Crit Care Med.

[22] Haase VH, Colgan SP, Karhausen J (2005). Inflammatory hypoxia. Cell Cycle.

[23] Gleich GJ (2000). Mechanisms of eosinophil-associated inflammation. J Allergy Clin Immunol.

[24] Nissim Ben Efraim AH, Eliashar R, Levi-Schaffer F (2010). Hypoxia modulates human eosinophil function. Clin Mol Allergy.

[25] Kopterides P, Kapetanakis T, Siempos II, Magkou C, Pelekanou A, Tsaganos T, Giamarellos-Bourboulis E, Roussos C, Armaganidis A (2009). Short-term administration of a high oxygen concentration is not injurious in an *ex-vivo* rabbit model of ventilator-induced lung injury. Anesth Analg.

[CR26] The pre-publication history for this paper can be accessed here:http://www.biomedcentral.com/1471-2253/14/86/prepub

